# Cell wall modification in *tobacco* by differential targeting of recombinant endoglucanase from *Trichoderma reesei*

**DOI:** 10.1186/s12870-015-0443-3

**Published:** 2015-02-13

**Authors:** Holger Klose, Markus Günl, Björn Usadel, Rainer Fischer, Ulrich Commandeur

**Affiliations:** Institute for Molecular Biotechnology, RWTH Aachen University, Worringer Weg 1, 52074 Aachen, Germany; Institute for Botany and Molecular Genetics, RWTH Aachen University, Worringer Weg 3, 52074 Aachen, Germany; Institute of Bio- and Geosciences, IBG-2: Plant Sciences, Forschungszentrum Jülich, Leo- Brandt-Straße, 52425 Jülich, Germany; Fraunhofer Institute for Molecular Biology and Applied Ecology (IME), Forckenbeckstrasse 6, 52074 Aachen, Germany

**Keywords:** Transgenic plant, Mesophilic endoglucanase, Cell wall modification, Differential targeting, *Nicotiana tabacum*, *Trichoderma reesei*

## Abstract

**Background:**

The development of transgenic plants as a production platform for biomass-degrading enzymes is a promising tool for an economically feasible allocation of enzymes processing lignocellulose. Previous research has already identified a major limitation of *in planta* production such as interference with the structure and integrity of the plant cell wall resulting in a negative influence on plant growth and development.

**Results:**

Here, we describe the *in planta* expression of endoglucanase TrCel5A from the mesophilic fungus *Trichoderma reesei* with differential intracellular targeting and evaluate its impact on the tobacco cell wall composition. Targeting of the enzyme to the apoplast leads to distinct changes in cell polysaccharides such as glucose level in the matrix polysaccharides (MPS). These effects are combined with severe changes in plant development. Retention of TrCel5A in the *endoplasmic reticulum* (ER) could avoid visible effects on plant growth under the chosen conditions, but exhibits changes in the composition of the MPS.

**Conclusions:**

These results give new insights into the complex interaction of heterologous cellulase expression with cell wall development and it outlines novel promising strategies to engineer plant cell walls for improved biomass processing.

**Electronic supplementary material:**

The online version of this article (doi:10.1186/s12870-015-0443-3) contains supplementary material, which is available to authorized users.

## Background

The utilization of lignocellulose, one of the most abundant renewable resources on earth, has the potential to play an important role in energy and commodity supply [1,2]. Structural polysaccharides, as part of the lignocellulose and therefore the plant cell wall, represent an extensive source of fermentable carbohydrates [3]. These polysaccharides, consisting of cellulose, hemicellulose and pectin, form a highly cross linked network providing the cell shape tensile properties and hence the structural stability of the plant [4]. These characteristics are also responsible for a major drawback in enzymatic lignocellulose utilization. Plant cell walls have evolved the ability to resist the enzymatic attack from microbes [5]. Therefore, without additional pretreatment the conversion of plant cell walls to fermentable sugar is comparatively slow and economically unfeasible [5]. To establish economically feasible processes utilizing lignocellulose, the recalcitrance of this substrate has to be overcome [6]. Appropriate modification of plant cell wall polymers can be a promising way to increase the feasibility of plant biomass utilization [7]. Plant cell wall synthesis is regulated by both biosynthesis and degradation with an extensive number of genes involved [8]. Cellulose, the crystalline structure of the microfibrills is synthesized by the cellulose synthase modules (CesA) in the plasma membrane [9,10]. In contrast, the synthesis of hemicellulose and pectin begins in the Golgi bodies [11] followed by transport to the apoplastic space where further modification occurs. These are then incorporated into the matrix phase of the cell wall fibers [12,13].

Glycosyl hydrolases (GHs) e.g. endoglucanases (EGs) play an important role in development, remodeling and degradation of different organisms’ cell wall mainly plants but also for example fungi. Plant GHs are mostly involved in fiber matrix remodeling or in cell wall plasticity during growth and development of the plant [14-16]. Biomass decomposing microorganisms like fungi or *Actinobacteria* produce these enzymes to convert plant cell wall polysaccharides into monosaccharides for their own metabolism [17].

The recombinant expression of GHs *in planta* has been attempted for different reasons, low-cost enzyme production [18,19], modification of starch [20,21] and reducing the recalcitrance of cell walls [22-24].

Promising examples have been described for *Acidothermus cellulolyticus* endoglucanase E1 in tobacco and maize which resulted in an improved conversion rate of the plant material [23]. Similar advantages have been described for endoxylanase 229B from *Dictyoglomus thermophilum* [24]. However, already described approaches did not allow a direct comparison between enzymatic effect and plant phenotype, e.g. cell wall structure and degradability [22]. Therefore, additional research with systematic analysis is needed.

Recombinant GHs have been targeted to various subcellular compartments with different results in expression level, stability and impact on the plant growth and development [25-27]. Thermophilic GHs have been found to be expressed with no harmful effect to the plant due to their limited activity at low temperatures [28-30]. Also sequestration by differential targeting and therefore limiting the access of hydrolytic enzymes to the plant cell wall has been addressed but with different effects. E.g. expression of *Aspergillus niger* ferulic acid esterase in *Festuca arundinacea* (tall fescue) with localization in ER or Golgi apparatus did show free mono- and dimers of ferulic acid and hence a higher degradability of the plant cell wall [31-33].

Here, we compare the heterologous expression of a mesophilic cellulase from *Trichoderma reesei* targeted to the ER and apoplast. We demonstrate a correct localization combined with high level expression of the active enzyme in both subcellular compartments. Furthermore, we analyze and correlate the biochemical phenotype of the cell wall derived polysaccharides of both localization variants and evaluate their differences relevant for a subsequent hydrolysis.

## Results

### Transgenic tobacco plants with different TrCel5A localization

In order to study the impact of TrCel5A expression with differential subcellular localization, tobacco plants with two different subcellular localizations were analyzed. Tobacco lines expressing TrCel5A localized in the apoplast were taken from a previous study [34].

For plants retaining TrCel5A inside the ER, the endoglucanase gene was fused to the sequence for a C-terminal KDEL signal (Additional file 1 Supplemental figures). Successful cloning was verified by transient expression in *N. tabacum* [35] followed by the detection of the enzyme by Western blot (Additional file 1 Supplemental figures).

Constructs encoding the enzyme with and without the KDEL tag were introduced into tobacco (*Nicotiana tabacum* SR1) leaf discs by Agrobacterium-mediated transformation [36]. Each generation of transgene plants was screened for the presence of the enzyme by Western blot. Transgene integration was confirmed by genomic PCR and the enzymatic activity was tested by the conversion of 4-methylumbelliferyl β-D-cellobioside (4MUC) to 4-methylumbelliferon (4MU) and cellobiose (data not shown). Homozygous lines revealing a 3:1 segregation ratio consistent with a single locus insertion were used to produce subsequent generations of plants (Table 1).

**Table 1:** **Expression of TrCel5A in transgenic tobacco**

**Construct**	**Gene**	**Subcellular localization**	**Total plants**	**Number of events**	**Expression level T1**	Expression level T2*
**TrCel5A**_**ER**_	AAA34213.1	ER	50	39	15.9 ± 11.8	40.8 ± 5.2
**TrCel5A**_**AP**_	AAA34213.1	Apoplast	35	13	9.4 ± 7.5	29.9 ± 6.4

Events and plants that produced seed from vectors designed to express endoglucanase from *T. reesei*. The mean value of expression level [nmol 4MU min^−1^ mg^−1^] of all positive plants for each construct is shown. *Values for second-generation represents the mean of three homozygous lines (T2) with ten samples each are also shown.

**Figure 1:**
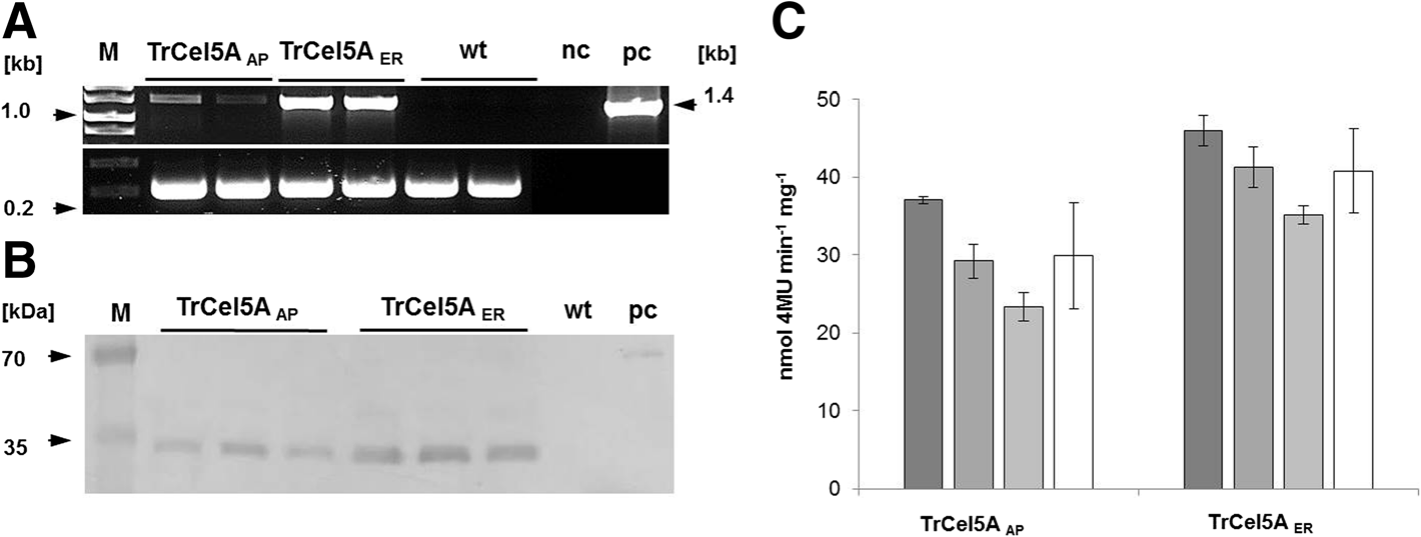
**Generation of transgenic tobacco expressing TrCel5A.** Genomic PCR of DNA prepared from wt and transgenic plants with primers binding in the CaMV 35SS expression cassette and below genomic PCR with primers binding in a highly conserved region in the chloroplast genome [38] **(A)**; M, GeneRuler™ 1 kb wt: SR1 wild type, nc: H_2_O, pc: control plasmid DNA pTRAkc-TrCel5A-ER. Western Blot for recombinant TrCel5A from three different TrCel5A_AP_ and TrCel5A_ER_ lines **(B)**. Lanes contain 10 μg of TSP from transgenic plants; Antibody system used in is MαHis5 /GAM_Fc_^AP^; wt: SR1 wild type, pc: purified His6 tagged Phenylammonium lyase from *Zea mays* (ZmPAL-His6). In all transgenic lines the degraded TrCel5A is detectable. Additionally in TrCel5A_ER_ lines also small amounts of the full enzyme are detectable. Expression level of TrCel5A in transgenic tobacco lines was determined by conversion of 4MUC **(C)**. Three independent lines with five plants each were tested (coloured bars). White bars represent the average expression level in TrCel5A_AP_ and TrCel5A_ER_ lines respectively.

Western blots showed that homozygous transgenic plants expressing the secreted version of TrCel5A yielded a recombinant protein with a molecular weight of ~35 kDa, similar to that of the native catalytic domain (Figure 1B) consistent with our previous findings that the enzyme undergoes proteolytic cleavage in plant cells [34]. In contrast, homozygous lines expressing TrCel5A with a KDEL tag revealed two fragments. The lighter but more prominent had a molecular weight of approximately 35 kDa, similar to the weight of the catalytic domain [37] while the heavier fragment with approximately 42 kDa corresponds to the molecular weight of the holoenzyme. Recombinant protein expression levels in transgenic tobacco plants were determined by measuring the activity of total soluble proteins (TSP) against the substrates azoCMC and 4MUC. T2 plants expressing the secreted version of TrCel5A achieved a specific enzyme activity of up to 1.5 U mg^-1^ TSP on azoCMC (data not shown) and 35 nmol 4MU min^-1^ mg^-1^ on 4MUC (Figure 1C), whereas the ER-localized version achieved a specific enzyme activity of up to 2.1 U mg^-1^ on azoCMC and 46 nmol 4MU min^-1^ mg^-1^ on 4MUC (Figure 1C).

### Subcellular localization of TrCel5A in tobacco plants

**Figure 2:**
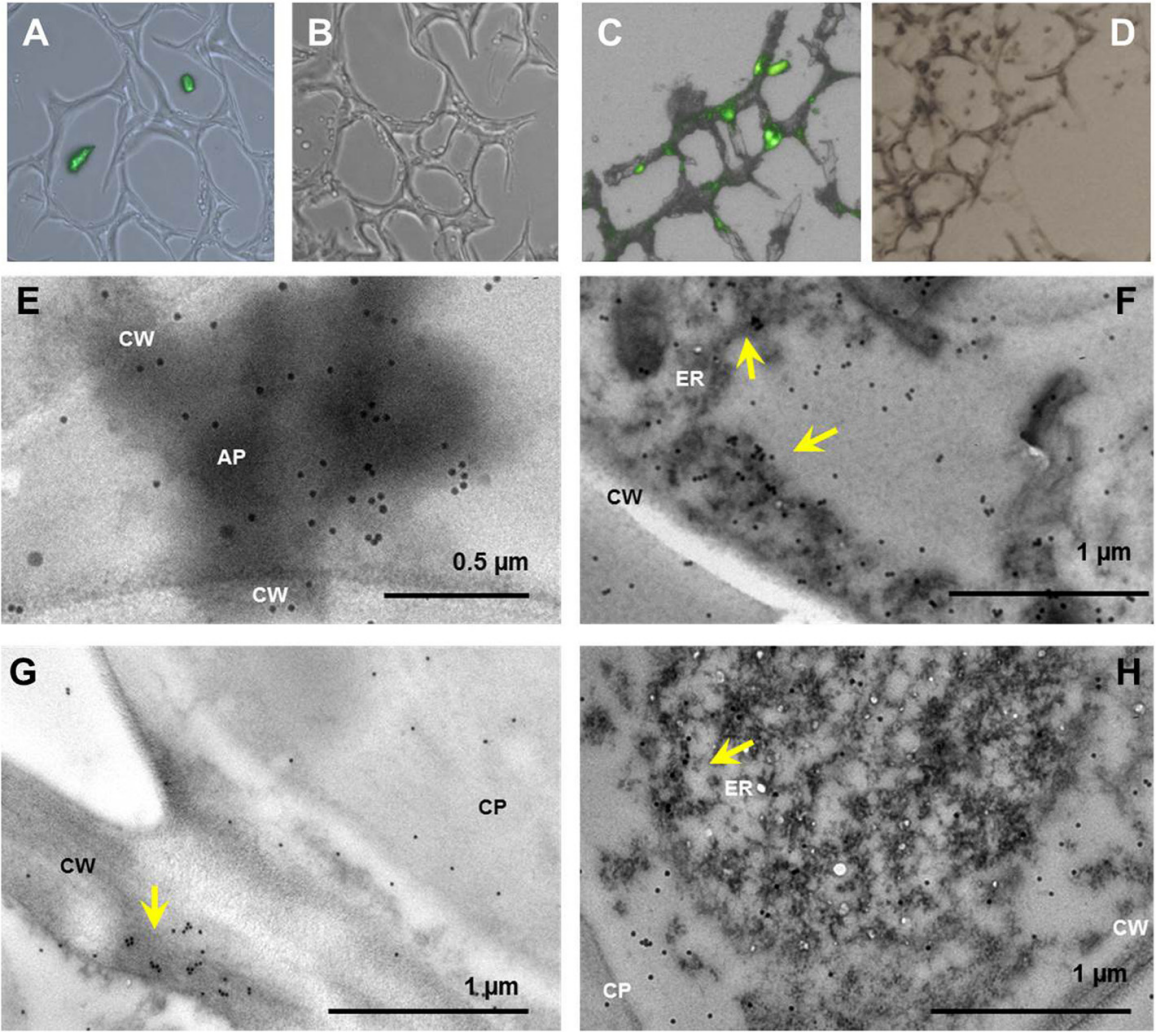
**Localization of TrCel5A in transgenic tobacco leaf tissue.** Plant tissue expressing TrCel5A targeted to the ER **(A)** and wt **(B)** were immunostained with the MαKDEL primary antibody and GAM_FC_^FITC^. Apoplast targeting was monitored by staining transgenic plant tissue and wt with RαCell primary antibody and GAR_FC_^FITC^ **(C-D)**. Electron microscopy with immunolabeling (RαCell/GAR_FC_^Gold^) indicated localization for TrCel5A_AP_ inside the apoplastic space or at the cell walls **(E, G)** and for TrCel5A_ER_ inside the ER **(F** **,** **H)**. Arrows mark accumulation of recombinant enzyme.

Localization of TrCel5A was examined in tobacco leaf tissue either by immunostaining with subsequent fluorescence or electron microscopy. Stained with either RαCell and GAR_FC_^FITC^ or MαKDEL and GAM_FC_^FITC^ a strong green fluorescence indicated the recombinant enzyme in the transgenic tissue, while no signal was detected in the wild type (wt) control line (Figure 2A-D). Stained with RαCell and GAR_FC_^Gold^ localization of TrCel5A was confirmed for apoplast and ER by electron microscopy (Figure 2E-H).

### Growth characteristics

**Figure 3:**
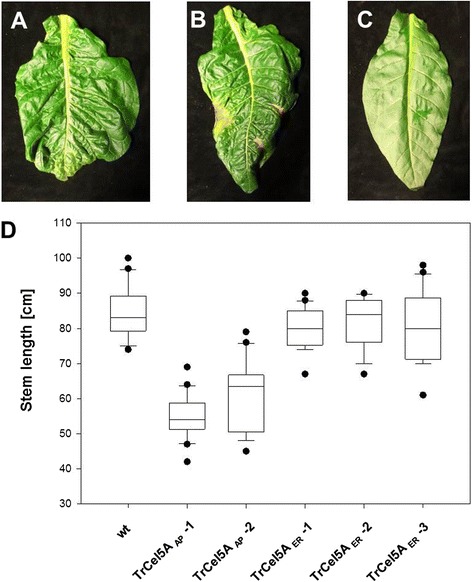
**Transgenic tobacco plants expressing TrCel5A.** Eight-week-old *N. tabacum* SR1 wt plants were grown in soil under standard conditions. Twenty plants of each line and wt were monitored on their stem size TrCel5A_AP_ plants showed a significant reduction in size and growth rate, whereas TrCel5A_ER_ plants were not significantly different from wt **(D)**. The leaf shape of several transgenic plants was altered compared to wt **(C)**. TrCel5A_ER_ **(A)** and TrCel5A_AP_ plants **(B)** displayed a curly leaf phenotype which in the case of TrCel5A_AP_ plants included additional necrotic lesions.

The constitutive expression of TrCel5A and its localization in the apoplast (TrCel5A_AP_) reduced the growth of tobacco plants and significantly delayed their development as described previously [34]. The stem length and germination rate were both reduced. The expression of TrCel5A with a KDEL peptide for retention in the ER (TrCel5A_ER_) resulted in a less significant difference in growth and development (Figure 3). For both constructs, a few transgenic plants displayed a curly and asymmetrical leaf phenotype, and in the TrCel5A_AP_ plants this was accompanied by necrotic areas on the leaf (Figure 3A and B).

### Chemical analysis of tobacco cell walls

**Figure 4:**
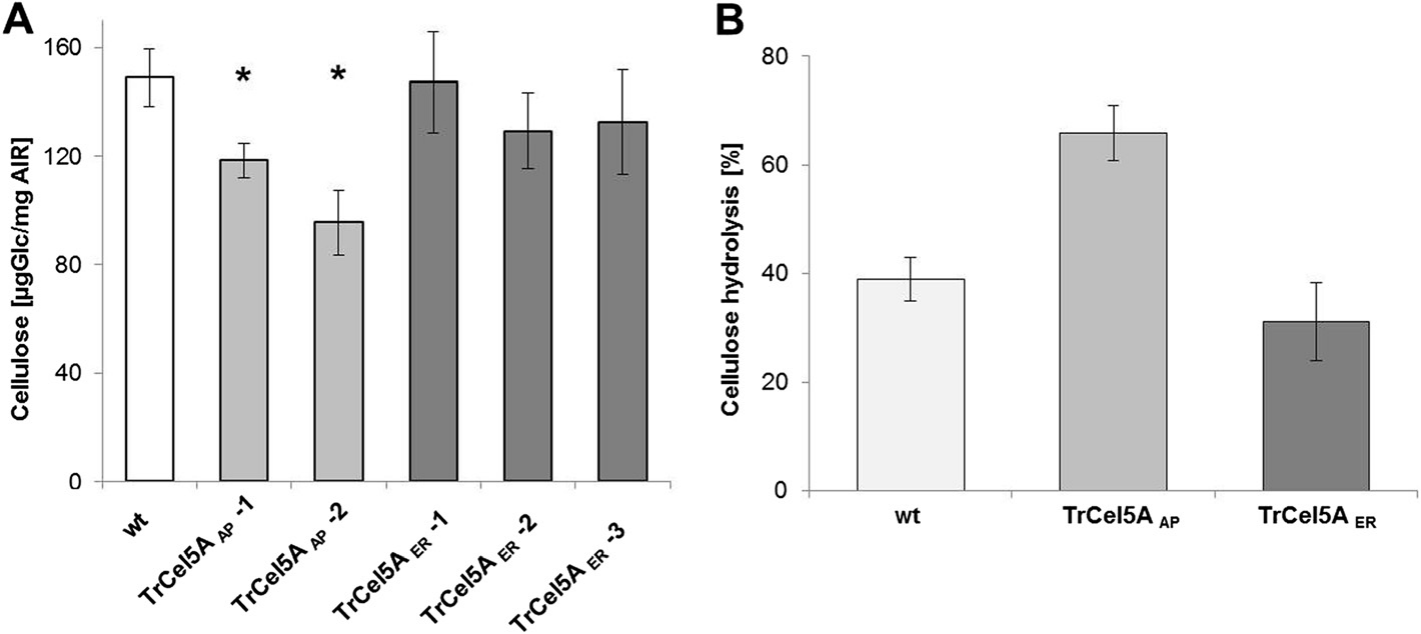
**Cellulose content and cell wall convertibility in transgenic tobacco.** The crystalline cellulose content and stem length was analyzed in six-week-old wt and TrCel5A transgenic tobacco plants grown in soil. Asterisks indicate a significant decrease (P ≤ 0.01) in the crystalline cellulose content for TrCel5A_AP_ compared to wt plants whereas TrCel5A_ER_ plants show no significant difference **(A)**. Leaf tissue from transgenic tobacco lines TrCel5A_AP_ and TrCel5A_ER_ **(B)**. Samples from both lines expressing ~30 mU/mg TSP (based on 4MUC conversion) were hydrolyzed with the appropriate amount of the commercial cellulase preparation [24]. Digestion was carried out at 55°C with constant shaking at 1000 rpm. The TrCel5A_AP_ leaves showed higher hydrolysis levels than the TrCel5A_ER_ and wt leaves after 8 h.

The structural carbohydrate composition of plants expressing differentially-targeted TrCel5A was determined in leaf material from six-week-old tobacco plants. Destarched alcohol-insoluble residue (AIR) was hydrolyzed with weak acid [39] and the solubilized carbohydrates were measured as alditol acetate derivatives by GC/MS [40]. The crystalline cellulose content of the tobacco leaf material was determined by Updegraff hydrolysis [41] and the anthrone cellulose assay. Transgenic lines expressing TrCel5A in the apoplast and ER were compared with wt. For transgenic lines accumulating TrCel5A in the apoplast, we could confirm the previous findings showing dramatic changes in the content of crystalline cellulose, which was significantly lower than in wt plants [34]. In contrast, transgenic plants accumulating TrCel5A in the ER did not show such differences (Figure 4A). In addition, the analysis of matrix phase polysaccharides revealed dramatic differences in the monosaccharide composition (Table 2). The TrCel5A_AP_ lines contained less glucose and more xylose. The TrCel5A_ER_ lines showed similar but smaller differences in these sugar levels than the TrCel5A_AP_ lines compared to wt levels. The amount of galactose increased in all transgenic lines. Arabinose and rhamnose levels were lower in the TrCel5A_AP_ lines but no change was found in the level of mannose. Two of three TrCel5A_ER_ lines contained lower levels of xylose, and all three lines contained slightly lower levels of mannose compared to wt plants. A significant reduction in the levels of arabinose and rhamnose was found in only one of the TrCel5A_ER_ lines. The biochemical changes in the cell wall sugars of the TrCel5A_AP_ lines were associated with a growth reduction phenotype, but as discussed above there was no significant visible change in the TrCel5A_ER_ lines despite the modulation of sugar levels. In addition to our previous findings [34], significant changes in the composition of matrix phase polysaccharides could be observed in transgenic lines with TrCel5A targeted to the apoplast. In contrast, transgenic tobacco plants targeting TrCel5A to the ER did not show these differences in crystalline cellulose content but also exhibit changes in matrix phase sugar composition.

**Table 2:** **Compositional analysis of matrix**-**phase polysaccharides in different transgenic tobacco lines**

**Line**	**Arabinose**	**Glucose**	**Galactose**	**Mannose**	**Rhamnose**	**Xylose**
**wt**	20.4	23.6	16,8	6.1	17.9	13.9
	±2.6	±8.4	±2.1	±1.1	±2.3	±1.8
**TrCel5A**_**AP**_ -**1**	14.9**	10.8* *	32.7**	5.3	14.4*	21.8**
	±1.4	±1.3	±6.7	±1.6	±3.68	±3.44
**TrCel5A**_**AP**_ -**2**	16.6*	13.9* *	34.1**	5.0	13.4**	16.9**
	±1.9	±4.7	±4.7	±1.2	±1.7	±1.9
**TrCel5A**_**ER**_ -**1**	20.7	17.9*	27.7**	5.0	16.9	13.9
	±1.7	±2.5	±2.9	±0.7	±1.6	±1.4
**TrCel5A**_**ER**_ -**2**	16.1**	19. 7*	33.1**	4.7*	13.4**	11.0**
	±1.1	±2.6	±2.4	±1.6	±1.1	±1.1
**TrCel5A**_**ER**_ -**3**	18.9	20.5*	27.2**	4.5*	16.2	12.0**
	±1.9	±3.4	±3.5	±1.4	±1.9	±1.2

Relative values [%] for sugar distribution in matrix polysaccharide fraction. Data analyzed by two-sided Student’s t-test; **indicates a significant P < 0.01; *indicates a significant P < 0.05; n = 20.

### Hydrolysis of leafy biomass from transgenic tobacco

To determine the hydrolysis rate of transgenic leaf material, tobacco plants were selected with similar TSP and TrCel5A activity levels (release of 4MU). Different Transgenic lines with both localizations were compared to wt plants. After enzymatic hydrolysis with a commercial cellulase cocktail (Novozymes, Bagsvaerd, Denmark), the remaining crystalline cellulose was determined from the insoluble remnants. In comparison to wt, transgenic leaf material with TrCel5A_ER_ did not show any significant change in the released relative cellulose values or the rate in hydrolysis, independent from the TrCel5A expression level. In contrast, leaf biomass from TrCel5_AP_ lines showed a significantly accelerated hydrolysis after 8 h (Figure 4). However, after 65 h the relative amount of solubilized crystalline cellulose (80 ± 1%) did not differ from TrCel5A_ER_ (79 ± 3%) and wt plants (77 ± 3%).

## Discussion

Current research in the area of biofuels aims to achieve the economically feasible production of cellulolytic enzymes for the conversion of lignocellulosic biomass into soluble sugars. Plants offer a relatively inexpensive production platform for industrial proteins [42,43] and therefore plants have been used to express a number of biomass-degrading enzymes [25-27].

Several studies have demonstrated the potential of *in planta* recombinant cellulases by achieving the accumulation of functional enzymes at high levels [18,23,44]. However, several drawbacks are associated with constitutive cellulase expression, including growth inhibition, changes in leaf morphology, loss of cell wall integrity and reduced stress tolerance [25,44,45].

Plants express endogenous GHs that play an important yet barely understood role in cell wall synthesis [46]. Both the overexpression and silencing of these GHs can have a negative impact on growth and cell wall development [14,47].

These issues have been addressed by targeting cell wall-degrading enzymes to different subcellular compartments, which can increase the overall enzyme yields [29,33,48] as well as keeping potentially harmful enzymes away from the cell wall [49,50]. We therefore established transgenic tobacco lines expressing the *T. reesei* mesophilic endoglucanase TrCel5A retained in the ER and compared them with transgenic lines from a previous study secreting TrCel5A to the apoplast [34].

For transgenic plants, TrCel5A localization was confirmed by immunostaining thin tissue sections, though the staining did not reflect the expression level of the enzyme.

As already described, TrCel5A underwent proteolytic cleavage within the linker region when expressed in tobacco [34]. However, the truncated enzyme remains active against soluble substrates such as azoCMC or other β-glucans [34,37]. Comparing the two localization strategies, the apoplast targeting yielded the truncated form of TrCel5A alone, whereas retention in the ER yielded a mixture of the truncated and full-length enzyme, although the latter was a minor product. TrCel5A activity in the transgenic plants was determined by measuring the conversion of 4MUC into 4MU, resulting in a maximum of 33 nmol 4MU min^1^ mg^-1^ in the TrCel5A_AP_ plants and 46 nmol 4MU min^-1^ mg^-1^ in the TrCel5A_ER_ plants. This probably reflects the relative scarcity in the number and quantity of proteases in the ER lumen compared to the apoplast [51].

As described previously, TrCel5A showed remarkable residual activity under physiological conditions typical for tobacco plants (20–30°C, pH 5.0) suggesting it potentially interferes with the synthesis of cell wall cellulose during normal plant growth and development [34]. Affirming our precedent findings, the TrCel5A_AP_ transgenic plants grew more slowly, germinated less frequently and showed a significant decrease in the amount of crystalline cellulose then wt. Despite the higher expression level of TrCel5A retained in the ER, no significant difference between TrCel5A_ER_ and wt were detected for these traits. Endoglucanase expression targeted to the apoplast has the potential to interfere with endogenous cellulose synthesis, where ER retention should avoid it, because the cellulose synthesis takes place in the plasma membrane by the cellulose synthase complexes. After polymerization, the glucan chains are extruded into the extracellular space [9]. This corresponds to our observation that the content of crystalline cellulose is reduced in TrCel5A_AP_ plants but not in TrCel5A_ER_ plants. However the role of endoglucanases during the synthesis of cell wall cellulose appears far more intricate. Plant endoglucanases such as KORRIGAN are necessary for the accumulation of sufficient cellulose and its absence leads to a cellulose deficiency and growth defects [14,52]. Recent studies have shown that the overexpression of KORRIGAN and other recombinant endoglucanases can also reduce the crystallinity of cellulose [23,34,47].

The analysis of matrix polysaccharide composition of transgenic and wt tobacco plants revealed a significant reduction in the relative amounts of glucose, arabinose and rhamnose in the TrCel5A_AP_ lines, but much higher levels of galactose and xylose. Plant GHs have been shown to remodel cell wall polysaccharides in the apoplastic space and during the transport of polysaccharide precursors through the Golgi apparatus [15]. Therefore, TrCel5A potentially interferes with the syntheses of Xyloglucan (XyG). However, TrCel5A is a strict endoglucanase and is only known to cleave β1,4 linkages between glucopyranose units, while showing no activity against complex mixed glucans [37]. Thus, the degradation is likely to take place at an earlier stage of XyG synthesis, when the glucan chains are still undecorated. The higher levels of galactose and xylose would directly reflect these losses of glucose.

The relative level of glucose was also lower in the TrCel5A_ER_ lines, albeit not to the extent seen in the TrCel5A_AP_ lines. Galactose levels were also higher, as in the TrCel5A_AP_ plants, but xylose levels were lower in two of three tested lines. All TrCel5A_AP_ plants had lower levels of mannose and one line also showed lower levels of arabinose and rhamnose. These changes in the matrix polysaccharide composition for TrCel5A_ER_ lines were not associated with a visible phenotype in contrast to the observed changes in the TrCel5A_AP_ plants.

ER localization was used to prevent the enzyme from entering the Golgi apparatus where most hemicellulose and pectin synthesis takes place [53,54]. However, recent studies have shown that ER-localized fungal enzymes can still affect matrix polymer synthesis. This has been shown for the deferuloylation of arabinoxylans in *F. arundinacea* by using a fungal ferulic acid esterase A. Even if the detected enzyme values for Golgi apparatus localization - the place of the synthesis - were higher, significant cleavage was observed for ER retardation of the enzyme [33]. This may reflect the promiscuous behavior of KDEL-tagged proteins, which can in some cases enter the Golgi network from where they are later retrieved [55].

XyG is the most abundant hemicellulose in the primary cell walls of dicotyledonous plants, representing up to 30% by weight [12]. In most dicots the main form is fucogalacto-XyG, but arabinogalacto-XyG is more abundant in solanaceous plants and monocots [13,56]. In fucogalacto-XyG, galactose is transferred to the xylosyl residue and then substituted with fucose, whereas in arabinogalacto-XyG galactose or arabinose can be transferred to the xylosyl residue [13]. The scarcity of glucose could then lead to the reduced levels of xylose in the matrix polysaccharides of TrCel5A_ER_ plants. In contrast, xylose levels were normal in the TrCel5A_AP_ lines. Interestingly, galactose levels were elevated in all the plants. Despite these differences in biochemistry, there was no significant morphological or developmental change in the TrCel5A_ER_ plants, which is consistent with previous observations of mutant plants with changes in the XyG side chain composition [57].

The remaining amount of crystalline cellulose was measured in the tobacco leaves after digestion with a commercial cellulase preparation, revealing a two-fold reduction in TrCel5A_AP_ plants after 8 h compared to wt and TrCel5A_ER_ plants. After 65 h however, all three lines contained the same relative amounts of residual cellulose, showing that degradation was accelerated but not enhanced overall in the TrCel5A_AP_ plants. Quantitative effects were ruled out by carefully selecting transgenic plants of both lines with similar overall enzyme expression levels, and we found no overall difference between the transgenic and wt lines. Endoglucanases produced *in planta* have previously been shown to make the plant cell wall less recalcitrant to hydrolysis [23]. Accordingly, our results confirmed a significant reduction in the levels of crystalline cellulose in TrCel5A_AP_ plants followed by accelerated hydrolysis.

The altered matrix polysaccharide fraction of the TrCel5A_ER_ plants appeared to have little impact on the enzymatic hydrolysis of leaf material, which was similar to the profile in wt leaves. This may reflect the relatively simple assay substrate, which does not consider the impact of lignin due to its low abundance in leaves compared to stems and roots [58]. The modification of cell wall hemicellulose using hydrolytic enzymes can promote lignocellulose degradation by reducing the amount of crosslinking between polymers [22,24,33].

## Conclusions

The heterologous expression of the mesophilic endoglucanase TrCel5A had a significant impact on the composition of plant cell wall polysaccharides, although the precise changes were dependent on the subcellular localization of the enzyme. The secreted enzyme notably reduced the content of crystalline cellulose and inhibited both growth and germination, whereas keeping the enzyme in the ER only altered the matrix phase polysaccharide composition without visible effects on plant development, suggesting a more important role of the crystalline cellulose for growth and development. This study obtained further insights on the impact of GHs on the plant and especially the cell wall polysaccharides, beneficial in designing crops to contribute to novel sustainable and renewable resources.

## Methods

### Construction of plant expression vectors

Cloning of plant expression vectors was done according to the previously described procedure [34]. The gene of *T. reesei* Endoglucanase TrCel5A (EGR51020.1, EMBL-CDS) was amplified by PCR using the forward primer cel5A (5’ TCC ATG GCA CAG CAG ACT GTC TGG GGC 3’) and the reverse primer cel5A rv (5’ TGC GGC CGC CTT TCT TGC GAG ACA CG 3’). These primers avoided the first 21 codons of the gene which contain the fungal signal peptide. The purified PCR product was transferred to the pCR2.1 vector (Invitrogen, Darmstadt, Germany) by TA-cloning to generate the construct pCR2.1 TrCel5A. Following digestion with *Nco*I and *Not*I, the released cassette was then inserted into the *Nco*I *Not*I linearized vector pTRAkc-ER.

### In planta expression of TrCel5A

Agrobacterium tumefaciens strain GV3101::pMP90RK was transformed with the above described vectors [59] by electroporation [60]. Colonies were transferred to liquid YEP medium (1% (w/v) Bacto tryptone; 1% (w/v) yeast extract; 0.5% (w/v) NaCl; pH7.0) containing kanamycin (50 mg ml^-1^), rifampicin (50 mg ml^-1^) and carbenicillin (100 mg ml^-1^) for 36–40 h (26°C, 180 rpm) for selection. The suspensions were then supplemented with 10 μM acetosyringone, 10 mM MES (pH 5.6) and 10 mM glucose, and incubated for another 20 h. The OD_600_ of the culture was adjusted to 1.0 with 2× infiltration medium (0.86% (w/v) MS salts, 10% (w/v) sucrose, 0.36% (w/v) glucose, pH 5.6) and supplemented with 200 μM acetosyringone prior to incubation for 45 min at room temperature. Transgenic tobacco lines were generated (*N. tabacum* L. cv. Petit Havana SR1) using the leaf disc transformation method [36]. T0 plants were grown on Murashige-Skoog medium containing 100 mg/L kanamycin and 200 mg/L Claforan, and subsequently transferred to soil in the greenhouse. T1 lines showing Mendelian segregation consistent with a single locus insertion were used for all further assays.

### Light and electron microscopy

Thin sections of 10 μm from transgenic and wild type leaf material were prepared using a Leica Cryostat Microtome and incubated in acetone for 30 min at room temperature. The thin section were blocked in phosphate buffered saline (PBS) containing 1% (v/v) Goat serum (Sigma-Aldrich, Seelze, Germany) and subsequently incubated with the primary antibody (diluted (1:50) in PBS containing 1% (v/v) Goat serum) for 45 min. Following three washing steps in PBS containing 0.05% (v/v) Tween-20 (Roth, Karlsruhe, Germany), the thin section were incubated with the secondary antibody (diluted (1:200) in PBS containing 1% (v/v) Goat serum for 45 min). After three washing steps in PBS containing 0.05% (v/v) Tween-20, the sections were analyzed using a Leica microscope.

For electron microscopy, tobacco leaves were fixed in 4% (w/v) paraformaldehyde and 0.5% (v/v) glutaraldehyde in 0.1 M phosphate buffer (pH 7.4), and then embedded in resin. Sections showing silver interference were collected on copper grids [61]. The primary antibody (diluted 1:50 in PBS) was detected with secondary antibodies (diluted 1:200 in PBS) labeled with 10 nm gold particles. Subsequently ultra-thin sections were stained with 2% (w/v) aqueous uranyl acetate and observed using a Philips EM-400 transmission electron microscope (Philips, Eindhoven, The Netherlands).

### Protein extraction and purification

Transgenic leaves were ground in liquid nitrogen and homogenized in PBS; pH 7.0 supplemented with 1 mM phenylmethylsulfonyl fluoride (PMSF). The extract was centrifuged at 15,000 x g for 20 min at 4°C followed by filtration. The His6-tagged protein was purified from TSP by Ni–NTA agarose affinity chromatography (Qiagen, Hilden, Germany). Imidazole was removed using a Roti®Spin column (Roth, Karlsruhe, Germany) with a molecular weight cut off 10 kDa. Total protein levels were determined using the Bradford method [62] with bovine serum albumin (Roth, Karlsruhe, Germany) as the standard.

### Polyacrylamide gel electrophoresis, and western blot

Protein samples were separated by SDS-PAGE in a 12% polyacrylamide gel. For Western blot analysis, separated proteins were electro-transferred (60 min, 250 mA) to nitrocellulose membranes, blocked for 1 h at room temperature with 5% (w/v) skimmed milk in PBS, and then probed first with a monoclonal Penta His Antibody (Qiagen, Hilden, Germany) and then with a monoclonal alkaline phosphatase-conjugated goat anti-mouse antibody (Dianova, Hamburg, Germany). The signal was visualized with nitroblue tetrazolium chloride/5-bromo-4-chloro-3’-indolyphosphate p-toluidine salt (NBT/BCIP) (Roth, Karlsruhe, Germany).

### Endoglucanase assays

Endoglucanase activity in crude plant extract was determined by the conversion of 4-MUC into 4MU, as described previously [63]. Samples were run in triplicate, with each sample (1–5 μl) assayed in 100 μl buffer (50 mM sodium acetate, pH 4.8, 0.5 mM 4-MUC) in a 96-well plate. Plates were covered with adhesive lids to prevent evaporation and incubated for 60 min at 50°C. The reaction was stopped by adding 100 μl 0.15 M glycine, pH 10.0. The fluorescence was determined using Tecan Infinite M200 (excitation wavelength of 360 nm, emission wavelength 465 nm). Fluorescence data of endoglucanase activity from plants with both localization for TrCel5A were corrected by subtraction of the average data from wt crude plant extract (n = 3). Conversion rates were calculated from corrected data based on a series of 4MU standards (1–10 nM).

### Cell wall sugar analysis

Cellulose was extracted from leaf tissue taken from 6–7 week-old plants. Ten samples were taken from each transgenic line and from wt tobacco plants. Alcohol-insoluble residues were prepared from frozen leaf tissue by grinding 50 mg of sample to a fine powder under liquid nitrogen, and isolating the plant cell walls by washing with different organic solvents [39]. Starch was removed by enzymatic digestion with α-amylase and pullulanase (Sigma-Aldrich, Seelze, Germany). The remaining AIR was extracted with acetone, dried and weighed. Matrix polysaccharide composition was determined with GC/MS measurement according to Foster *et al*. [38]. The crystalline cellulose content was determined [41] after hydrolyzing the non-crystalline cellulose with acetic and nitric acid. The remaining crystalline cellulose residues were hydrolyzed with 72% (w/v) sulfuric acid allowing the remaining glucose to be measured using the anthrone assay [64].

Significant differences from wt were determined using a Student t-test (p value ≤ 0.01).

### Conversion of transgenic tobacco leaf material

To estimate the glucan content, tobacco leaf material was hydrolyzed in sulfuric acid and the carbohydrates were determined using the anthrone assay [64]. For the conversion of leaf material, 50–100 mg of fresh tobacco leaf tissue was ground to a powder in liquid nitrogen and resuspended in buffer (50 mM sodium acetate, pH 4.8) containing 15 Filter paper units (FPU) CTec2 (Novozymes A/S, Denmark) per g glucan. The reaction was terminated by boiling for 10 min and subsequently centrifugation at 10.000 × g. To determine the rate of hydrolysis, the remaining crystalline cellulose was measured according to the previously described methods. Values were calculated from three independent experiments, each containing three replicates.

### Availability of supporting data

The data sets supporting the results of this article are included within the article and its additional files.

## Additional file

Additional file 1:
**Supplemental Figure S1:** **Schematic presentation of the plant expression cassettes for differential targeting of TrCel5A.** The CaMV promoter (P35SS) and terminator signal (pA35S) are indicated in light blue. 5’-UTR of chalcone synthase (CHS), the His6 coding sequence (His6) is indicated in blue. The plant codon-optimized leader peptide (LPH) derived from the heavy chain of the murine mAb24 is depicted in green. LPH achieves the secretion of the recombinant protein to the apoplast (A). Additionally added C-terminal KDEL signal indicated in red retards the protein to the ER (B). Arrows label the binding site for primer to amplify the CaMV 35SS expression cassette. (C) Western blot analysis for transient expression of TrCel5A for ER and apoplast localization compared with catalytic domain of TrCel5A (cd). Supplemental Figure S2: Monitoring transgenic tobacco plant growth over time. Tobacco plants were grown in soil (Einheitserde® Typ ED73) with 16/8 h light/dark cycle (500 μmol s-1 m2, λ = 400–700 nm) and daily watering. Additional fertilizer (WUXAL, ~0,05% v/v) was used once after 40 days with the spilling water. Bars represent 20 cm length.

## Abbreviations

4MU: 4-Methylumbelliferone
4MUC: 4‑Methylumbelliferyl β-D-cellobioside
AIR: Alcohol insoluble residues
AP: Apoplast
BCIP: 5 Bromo-4-chloro-3’-indolyphosphate p-toluidine
CaMV: Cauliflower Mosaic Virus
CBM: Cellulose binding module
CMC: Carboxymethylcellulose
EG: Endoglucanase
ER: Endoplasmic reticulum
FPU: Filter paper unit
GC: Gas chromatography
GH: Glycosyl hydrolase
MES: 2-(N-morpholino) ethanesulfonic acid
MPS: Matrix polysaccharides
MS: Mass spectrometry
MS: Murashige and Skoog
NBT: Nitro-blue tetrazolium chloride
NTA: Nitrilotriacetic acid
PAGE: Polyacrylamide gel electrophoresisPBS: Phosphate buffered saline
PMSF: Phenyl-methylsulfonyl fluoride
SDS: Sodium dodecyl sulfate
TSP: Total soluble protein
UTR: Untranslated region
Wt: wild type
XyG: Xyloglycan
YEP: Yest extract peptone.
