# Supplementary material for: Cell wall modification in tobacco by differential targeting of recombinant endoglucanase from Trichoderma reesei
Source: BMC Plant Biol. 2015 Feb 13;15:54. doi: 10.1186/s12870-015-0443-3 (PMC4340609; doi:10.1186/s12870-015-0443-3)
Supplement: Additional file 1: — Supplemental Figure S1: Schematic presentation of the plant expression cassettes for differential targeting of TrCel5A. The CaMV promoter (P35SS) and terminator signal (pA35S) are indicated in light blue. 5’-UTR of chalcone synthase (CHS), the His6 coding sequence (His6) is indicated in blue. The plant codon-optimized leader peptide (LPH) derived from the heavy chain of the murine mAb24 is depicted in green. LPH achieves the secretion of the recombinant protein to the apoplast (A). Additionally added C-terminal KDEL signal indicated in red retards the protein to the ER (B). Arrows label the binding site for primer to amplify the CaMV 35SS expression cassette. (C) Western blot analysis for transient expression of TrCel5A for ER and apoplast localization compared with catalytic domain of TrCel5A (cd). Supplemental Figure S2: Monitoring transgenic tobacco plant growth over time. Tobacco plants were grown in soil (Einheitserde® Typ ED73) with 16/8 h light/dark cycle (500 μmol s-1 m2, λ = 400–700 nm) and daily watering. Additional fertilizer (WUXAL, ~0,05% v/v) was used once after 40 days with the spilling water. Bars represent 20 cm length. [file 12870_2015_443_MOESM1_ESM.pdf]

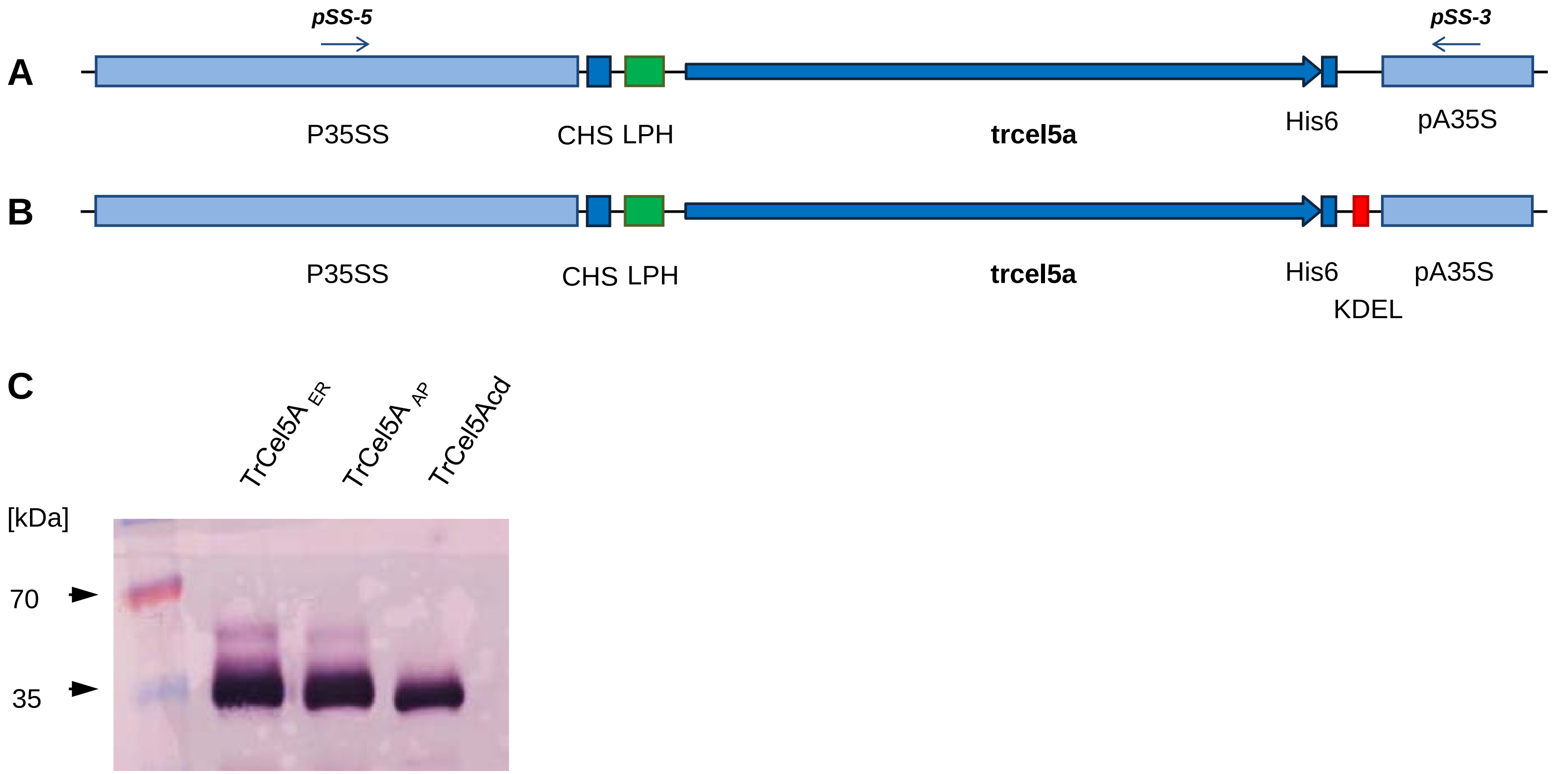

Supplemental Figure 1: Schematic presentation of the plant expression cassettes for differential targeting of TrCel5A. The CaMV promoter (P35SS) and terminator signal (pA35S) are indicated in light blue. 5'-UTR of chalcone synthase (CHS), the His6 coding sequence (His6) is indicated in blue. The plant codon-optimized leader peptide (LPH) derived from the heavy chain of the murine mAb24 is depicted in green. LPH achieves the secretion of the recombinant protein to the apoplast (**A**). Additionally added C-terminal KDEL signal indicated in red retards the protein to the ER (**B**). Arrows label the binding site for primer to amplify the CaMV 35SS expression cassette.
(**C**) Western blot analysis for transient expression of TrCel5A for ER and apoplast localization compared with catalytic domain of TrCel5A (cd).

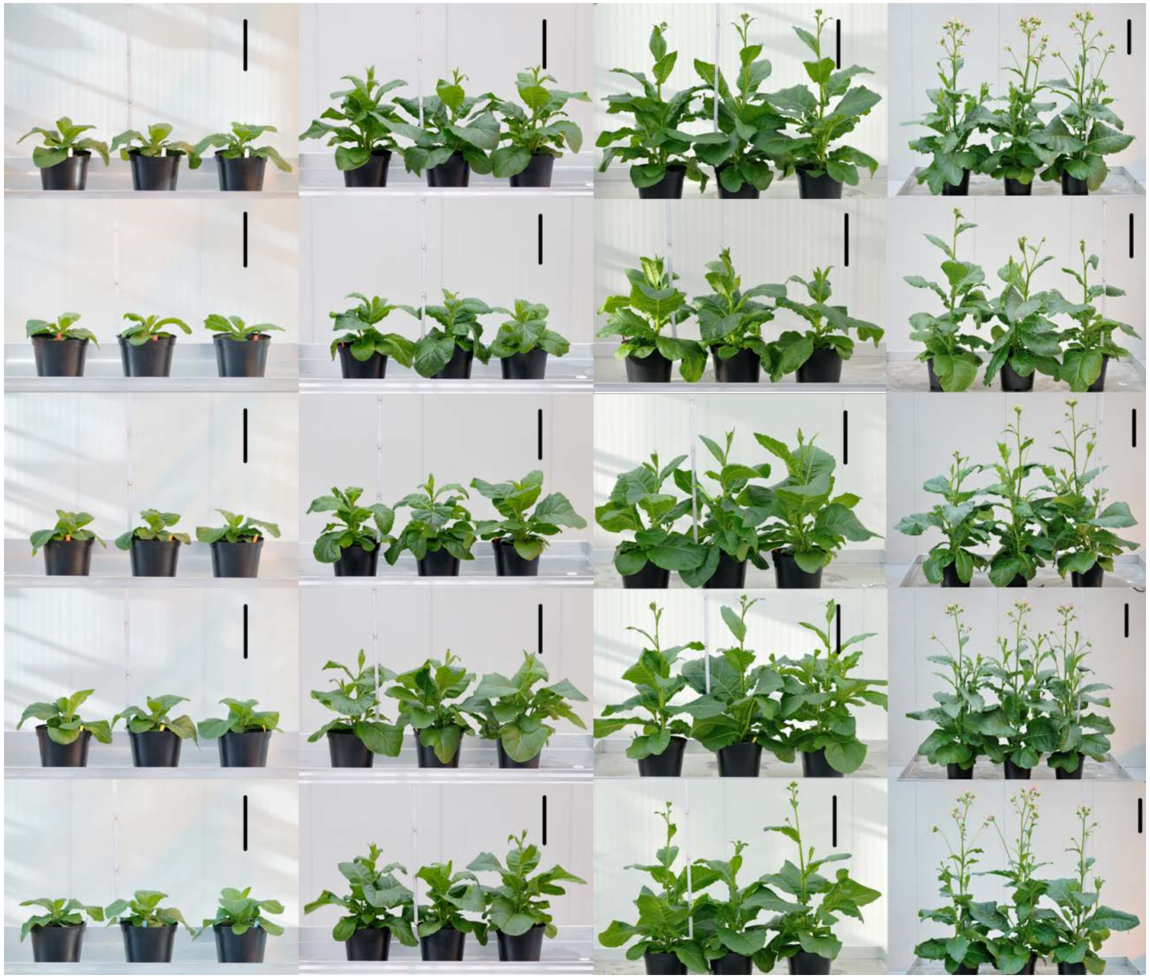

Supplemental Figure 2: Monitoring transgenic tobacco plant growth over time. Tobacco plants were grown in soil (Einheitserde® Typ ED73) with 16/8 h light/dark cycle (500 µmol s-1 m2, λ= 400-700 nm) and daily watering. Additional fertilizer (WUXAL, ~0,05% v/v) was used once after 40 days with the spilling water. Bars represent 20 cm length.
